# P-1677. Assessing An Institution’s Approach to Management of Gram-Negative Bacteremia in High-Risk Populations

**DOI:** 10.1093/ofid/ofae631.1843

**Published:** 2025-01-29

**Authors:** Anahita Mostaghim, Nehal G Hashem, Sabina Pathan, Alison Han

**Affiliations:** Atrium Health, Charlotte, North Carolina; NIH, Bethesda, Maryland; National Institute of Allergy and Infectious Disease, Bethesda, Maryland; National Institutes of Health, Bethesda, Maryland

## Abstract

**Background:**

Immunocompromised populations present unique antimicrobial management and stewardship challenges. These challenges can persist despite existing stewardship policies. This study examined management of gram-negative bacteremia (GN-BSI) in a diverse immunocompromised population at the National Institutes of Health Clinical Center (NIH CC).

**Figure:**
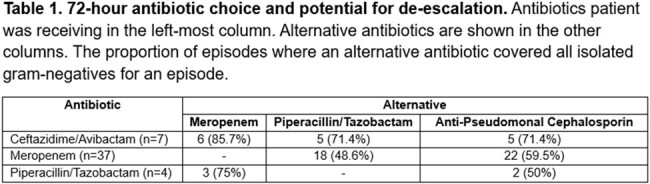

**Methods:**

Episodes of GN-BSI admitted to the NIHCC from January 1 to December 31, 2023 were identified through positive blood culture results. Charts were abstracted for baseline demographics, episode-specific data, antimicrobial prescribing, and clinical outcomes.

**Figure:**
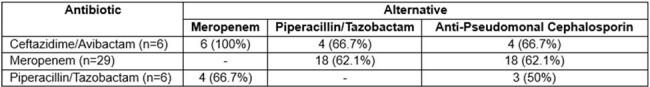

**Results:**

Seventy-five episodes of GN-BSI were identified. Thirty-six (48%) were undergoing cellular therapy and 54 (58.7%) were neutropenic. About 50.7% identified translocation as etiology of infection. Eleven (14.7%) were ESBL-positive by BCID2 panel. Seventy-two (96%) involved infectious disease (ID) consultation. Empiric choice of gram-negative antibiotics included ceftazidime/avibactam in 9 (12%) episodes, meropenem in 38 (50.7%) episodes, piperacillin/tazobactam in 15 (20%) episodes, and an anti-pseudomonal cephalosporin in 32 (42.7%). Twenty (26.7%) received 2+ empiric anti-pseudomonal antibiotics. After 72 hours, 37 (49.3%) still received meropenem with most isolates susceptible to narrower spectrum (Table 1). Similar results were seen with final ID recommendations (Table 2). Seven (10%) had transitioned to an oral agent by the seventh day. Comparing 72-hour antibiotic (Meropenem vs anti-pseudomonal cephalosporin), ICU admission (32.4% v 25%, p=0.62) and 30-day mortality (5.4% v 6.3%, p=0.87) were similar. Median therapy duration was 14 days (IQR 14-18.5). Of the 65 episodes with indwelling central venous access (CVAD), only 16 had removal of all CVADs. Those with CVAD removal had lower 30-d mortality (0% v 11.9%, p=0.22) though not statistically significant.

**Conclusion:**

In a high-risk immunocompromised population, there is a low rate of narrowing gram-negative coverage at 72 hours although options for narrowing exist. Choice of empiric antimicrobial and targeted therapy for these GN-BSI episodes are the two major areas of improvement in our population. Primary teams and ID consultants should be involved in ongoing stewardship interventions.

**Disclosures:**

**All Authors**: No reported disclosures

